# Psychiatric outcomes and long-term school and work-related disability in offspring of parents with depression and treatment-resistant depression

**DOI:** 10.1007/s00127-025-02988-z

**Published:** 2025-09-22

**Authors:** Philip Brenner, Heidi Taipale, Pontus Josefsson, Allitia DiBernardo, Antti Tanskanen, Ellenor Mittendorfer-Rutz, Johan Reutfors

**Affiliations:** 1 Centre for Pharmacoepidemiology, Department of Medicine Solna, Karolinska Institutet, Karolinska University Hospital, Stockholm, Sweden; 2 Centre for Psychiatry Research, Department of Clinical Neuroscience, Karolinska Institutet, & Stockholm Health Care Services, Region Stockholm, Sweden; 3 Division of Insurance medicine, Department of Clinical Neuroscience, Karolinska Institutet, Stockholm, Sweden; 4 Department of Forensic Psychiatry, Niuvanniemi Hospital, University of Eastern Finland, Kuopio, Finland; 5 School of Pharmacy, University of Eastern Finland, Kuopio, Finland; 6 Janssen Global Services, LLC, Titusville, NJ USA

**Keywords:** Major depressive disorder, Children and adolescents, Treatment resistant depression, Epidemiology, Antidepressants

## Abstract

**Purpose:**

To investigate the risk for adverse outcomes among offspring of parents with depression and with treatment-resistant depression (TRD), compared with matched offspring in the general population.

**Methods:**

Parents diagnosed with depression in specialized psychiatric care in 2006–2018 were identified in nation-wide Swedish registers. Those starting a third sequential antidepressant trial were defined as treatment-resistant. Parents and their 2,359 first-born offspring, aged 6–15 years when parents were defined with TRD, were closely matched 1:1 with parent-offspring pairs with other parental depression as well as with parent-offspring pairs from the general population. Offspring cohorts were followed prospectively for psychiatric outcomes and school- and work-related disability.

**Results:**

Offspring of parents with both TRD and other depression had substantially elevated risks for all outcomes compared to general population offspring. Adjusted hazard ratios for offspring of parents with TRD were: depression 4.6 (95%CI 3.2–6.5); contact with psychiatry 3.3 (2.8–4.0); psychiatric medication 3.6 (3.0–4.2); suicide attempt 3.2 (1.9–5.5); sick leave for mental health reasons 2.3 (1.1–4.6); and disability pension 4.2 (2.2–8.1). The adjusted odds ratio for non-completion of secondary school when expected was 2.1 (1.5–2.9). In direct comparisons between offspring of parents with TRD vs. other depression, relative risks for all outcomes were similar, with no statistically significant differences.

**Conclusion:**

Offspring of parents with TRD and other depression are at similarly elevated risks of adverse clinical, educational, and work-related outcomes. Parental TRD, as defined in administrative health care data, may not serve as a risk indicator for long-term offspring burden in parental depression.

**Supplementary Information:**

The online version contains supplementary material available at 10.1007/s00127-025-02988-z.

## Background

Having a parent with depression is associated with increased risks for various negative outcomes throughout life. While offspring of mothers with an ongoing depressive episode are at higher risk for mental disorders and behavioral difficulties during childhood [1], long-term follow-up studies have revealed higher risks for psychiatric, somatic, and societal consequences persisting well into adulthood [2–4]. For instance, perinatal parental depression is associated with an elevated risk of depression in the offspring by the age of 18 [5].

Regardless of whether the mother or father is affected, parental depression is linked to an increased risk of mental disorders in the offspring, as well as the disorders having a more severe course [6, 7]. Having a parent with depression is associated with a 40% increase in adolescents’ alcohol and nicotine dependence, irrespective of parental anxiety and substance use [6]. Parental depression is also a strong risk factor for suicidal behavior and suicide, and this may increase with the severity of the parental depression [8, 9].

Parental depression influences school performance and later occupational function as well. Both perinatal and later-life parental depression is associated with poorer school performance at age 16 [10], and maternal postnatal depression may be a risk factor for worse cognitive performance in offspring in school age [11]. Parental mental disorders are also risk factors for future work disability in offspring, mediated by a higher risk for mental disorders and adolescent social disadvantages [12].

Existing etiological models suggest shared biological and environmental causal pathways [13, 14], with some studies emphasizing the significance of the rearing environment [15, 16]. Some studies show maternal depression to be a stronger risk factor than depression in the father [15, 17], while others attribute equal importance to depression regardless of the parent’s sex [18].

The treatment or remission of the parental depression may affect the risk for adverse outcomes in the offspring. Offspring of mothers who reached remission in the Sequenced Treatment Alternatives to Relieve Depression (STAR*D) study had a lower risk of psychiatric or behavioral disturbances at follow-up [19]. Improvement in parents’ depression has also been associated with subsequent improvement in their children’s depressive symptoms and functioning [20]. However, longitudinal studies of clinical samples have also demonstrated that depression can be recurrent and chronic, with enduring negative impacts on offspring [4, 21].

The impact of a non-remitted parental depression on the offspring has not yet been investigated in large cohorts with long follow-up times. Remission of depression is a clinical concept that is challenging to measure in administrative datasets. The term treatment-resistant depression (TRD) is commonly used when two treatment trials for depression have not yielded adequate effects. Up to 50% of patients initiating antidepressant treatment have been classified as having TRD in clinical studies evaluating consecutive treatment trials [22, 23]. In register-based studies that employ pharmacoepidemiological algorithms to define TRD, the proportion is 11–21% [24–27]. Patients with TRD have an increased risk for various detrimental outcomes compared to other patients with depression, including long term work disability, hospitalization, and death [24, 28, 29].

Overall, there is a well-established link between parental depression and various negative outcomes for the offspring, with evidence from smaller cohort studies suggesting that remission of parental depression reduces the strength of this association. We hypothesized that offspring of parents with TRD would have a higher risk for negative psychiatric and work disability outcomes compared to offspring of other depressed parents and those with no parental depression. The aim of this study was to investigate this hypothesis using a pharmacoepidemiological model for TRD designed for use in Swedish national register data.

## Methods

### Data sources and study population

#### Cohort of parents with depression and antidepressant initiation

**Fig. 1:**
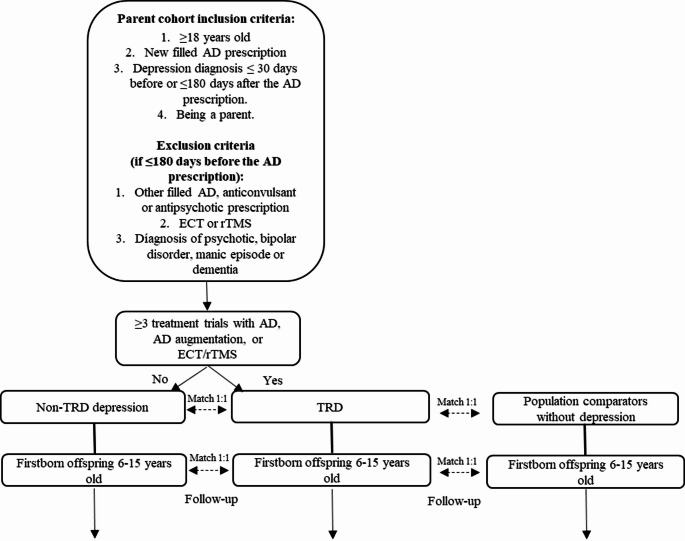
Study population selection flow-chart. AD = antidepressant, ECT = electroconvulsive therapy, rTMS = repetitive transcranial magnetic stimulation, TRD = treatment resistant depression

To identify parents with a new episode of depression, the following Swedish registers were utilized: the National Patient Register (NPR) [30], the Prescribed Drug Register [31], the Longitudinal Integrational Database for Health Insurance and Labor Market Studies (LISA) [32], the Multi-Generation Register [33] and the Total Population Register (TPR) [34]. Individuals 18 or older who met the following criteria were included: (a) filled a prescription for an antidepressant drug (AD; Anatomical Therapeutical Chemical [ATC] code N06A) without any previous AD dispensing in the preceding 180 days in Sweden between July 1, 2006, and December 31, 2018 (hereafter referred to as the index AD fill); (b) received a diagnosis of a depressive episode (International Statistical Classification of Diseases and Related Health Problems, 10th Revision [ICD-10] code F32 or F33) in specialist care within 30 days before or up to 365 days after the index AD fill; and (c) being a parent. For a flow-chart illustrating the cohort selection and subsequent matching procedure, see Fig. 1.

Exclusion criteria included any history of: a) an ICD-10 diagnosis of (a) dementia, (b) bipolar disorder or manic episode; or (c) psychotic disorders, as well as having filled a prescription in the 180 days preceding the index AD fill of (d) antipsychotics or lithium; or (e) anticonvulsants/mood stabilizers. For all ICD-10 codes and ATC codes used, see Suppl Table 1. Further, patients with the following measure codes in the NPR within 180 days before the index AD fill were excluded: (f) electroconvulsive therapy (ECT); (g) vagus nerve stimulation; and (h) repetitive transcranial magnetic stimulation (rTMS). Finally, i) patients who were not residents in Sweden in the two calendar years preceding the year of the index AD fill were excluded.

The commonly used definition of TRD, i.e. two adequate treatment trials without satisfactory response, was adapted for this study through an algorithm used in previous studies from our group, described in detail elsewhere [35]. Patients initiating a third treatment trial within 365 days from the index AD fill, using antidepressant monotherapy, combination therapy, add-on therapy with antipsychotics or mood stabilizers, or Electroconvulsive Therapy (ECT) or repetitive Transcranial Magnetic Stimulation (rTMS), were classified with TRD. The medication trial had to last at least 30 days according to package size and dosing texts.

#### Matching of offspring with general population comparators

From the cohort of parents with depression and antidepressant initiation, those parents were selected who fulfilled the TRD criteria when their oldest offspring was between 6 and 15 years of age. These parents and their oldest offspring formed TRD parent-offspring pairs. Each TRD parent-offspring pair was matched 1:1 with a parent-offspring pair from the general population in which the parent satisfied the following eligibility criteria for matching: (a) no record in the registers of an ICD-10 diagnosis of depression or any of the ICD-10 diagnoses listed as exclusion criteria for inclusion in the depression cohort above, (b) no ATC codes or measure codes corresponding to the exclusion criteria for the cohort of parents with depression and antidepressant initiation; and (c) resident in Sweden in the two calendar years preceding the year of the index AD fill of the TRD parent. The date of matching was the date of TRD criteria fulfillment. Matching criteria were: (a) age of the offspring at the matching date, (b) sex of the offspring (c) birth rank of the offspring, i.e. the oldest offspring, (d) age of the parent at the index AD fill date of the TRD parent, (e) sex of the parent, and (f) type of living area of the parent at the index AD fill date of the TRD parent (classified as cities, towns and suburbs, or rural areas).

#### Matching of TRD parent-offspring pairs to parent-offspring pairs with other parental depression

Additionally, each TRD parent-offspring pair was matched to a parent-offspring pair in which the parent was included in the cohort of parents with depression and antidepressant initiation but did not meet TRD criteria at the matching date, hereafter termed “other depression”. Matching criteria were: (a) age of the offspring at the matching date (± 1 year), (b) sex of the offspring, (c) birth rank of the offspring, i.e. the oldest offspring, (d) age of the parent at the index AD fill date (± 5 years), (e) sex of the parent, (f) type of living area of the parent at the index AD fill date, (g) year of the index AD fill date.

The age matching criteria were wider for depression comparator pairs due to a lower number of comparators available. All matching was exclusive, i.e. without replacement. An offspring could not be available for matching as a parent at a later date. If a parent comparator with other depression later fulfilled TRD criteria, he/she would be followed as such and matched with a new other depression comparator at that date. If both parents were available for cohort selection, only one parent was selected for each offspring. The offspring formed a pair with the parent who first fulfilled the TRD criteria. If none of the parents fulfilled the TRD criteria, the offspring formed a pair with the parent who first fulfilled the criteria for the cohort of parents with depression and antidepressant initiation. Each TRD parent-offspring pair formed a matched group together with its two 1:1 matched parent-offspring pairs.

#### Covariates

**Fig. 2:**
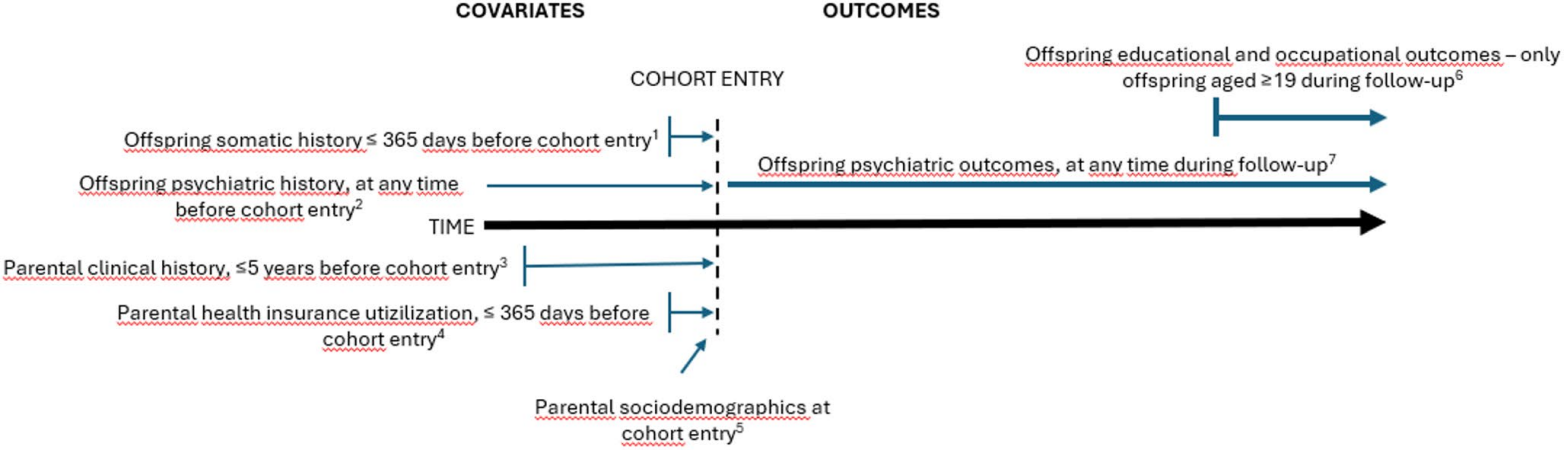
Time intervals for selection of covariates and outcomes. ^1^Any non-psychiatric healthcare visit. ^2^Any psychiatric diagnosis. ^3^History of substance use, depression, other psychiatric disorders or self-harm/suicide attempts. ^4^Number of sick leave days and/or disability pension. ^5^Country of birth, attained education level, family situation, number of children, latest available data at end of calendar year preceding cohort entry. ^6^Not having completed secondary school during the year when reaching 19 years of age and sick leave with psychiatric diagnosis and disability pension from July 1 in the calendar year when the offspring reached 19 years of age. ^7^Any psychiatric contact, depression diagnosis, psychiatric medication, suicide attempt, and suicide.

The analysis was adjusted for several covariates regarding parental demographics, parental clinical history, parental history of social benefits, and offspring clinical history. Parental demographics were (a) country of birth (Sweden, Europe other than Sweden, Rest of the world); (b) educational level according to the latest available information in the LISA database (< 9 years, 10–12 years, > 12 years); (c) family situation according to the latest information in the TPR at time of inclusion (Married or cohabitant without children, Married or cohabitant with children, Single household without children, Single household with children); and (d) number of children according to the latest information in the TPR at time of inclusion (1, 2, > 2). The variables describing educational level, family situation and number of children were based on register information from the end of the calendar year preceding the calendar year of the index AD fill. See Fig. 2 for a visualization of time frames for definition of covariates and outcomes.

Covariates regarding parental clinical history were (a) depression (No or Yes) based on main- and secondary ICD-10 diagnoses; substance use disorder (No, Alcohol only, Other/combined) based on main- and secondary diagnoses and filled prescriptions of drugs; (b) other major psychiatric disorders (No, Yes), defined as anxiety disorders, obsessive compulsive disorders, eating disorders, personality disorders, autism and attention deficit hyperactivity disorder based on main- and secondary diagnoses; (c) history of self-harm/suicide attempts (No, Yes) based on external cause codes; and (d) non-psychiatric healthcare use (No, Yes) based on main diagnoses. The variable describing depression was created based on information from the 5 years preceding the index AD fill date, excluding the 180 days wash-out period preceding the index AD fill date (1645 days), and the variables describing substance use disorder, other psychiatric disorders, self-harm and non-psychiatric healthcare use were created based on information from the 5 years preceding the index AD fill date (1825 days). All codes used are listed in Suppl Table 1.

Parental history of social insurance benefits were (a) sick leave days (0, 0–90, 90–365); and (b) disability pension (No, Yes). The variables describing sick leave days and disability pension were created based on net days with benefit from the social insurance agency in the year preceding the index AD fill date (365 days).

Offspring clinical history were (a) Psychiatric disorders (No, Yes) based on main- and secondary diagnoses; (b) self-harm (No, Yes) based on external cause codes; and (c) non-psychiatric healthcare use (No, Yes) based on main diagnoses. The variables describing mental and behavioral disorders and self-harm were created based on all available information from before the matching date, and the variable describing non-psychiatric healthcare use was created based on information from the year preceding the matching date (365 days).

### Outcomes

Four clinical and three socioeconomic outcomes were measured for the offspring. Offspring clinical outcomes were (a) overall contact with psychiatric care based on medical specialty codes; (b) depression diagnosis based on main- and side diagnoses; (c) psychiatric medication based on filled prescriptions of drugs; (d) suicide attempt based on external cause codes; and (e) suicide recorded as cause of death. Offspring socioeconomic outcomes were (f) not having completed secondary school in the year when reaching 19 years of age; (g) sick leave with psychiatric diagnosis; and (h) disability pension.

All clinical outcomes were measured as time to the event of interest from the matching date. Sick leave with psychiatric diagnosis and disability pension were measured as time to the event of interest from July 1 in the calendar year when the offspring reached 19 years of age. The following were censoring events: death, emigration, end of follow-up time (Dec 31 st 2018), or a parental comparator with other depression meeting TRD criteria. Not having completed secondary school in the year when aged 19 was measured as a binary variable (No, Yes) on Dec 31 st in the calendar year when the offspring reached 19 years of age. Measurement of the socioeconomic outcomes was restricted to offspring for whom none of the censoring events had occurred prior to July 1 st or Dec 31 st in the calendar year when the offspring reached 19 years of age.

### Statistical analyses

Separate analyses were conducted for each outcome, not taking the other outcomes into account. All outcomes, except not having completed secondary school at 19 years of age, were analyzed using stratified Cox regression, taking the matched design into account by allowing the baseline hazards to differ across matched groups. Follow-up started at the matching date, except for sick leave with a mental diagnosis and disability pension, for which follow-up started on July 1 in the calendar year the offspring turned 19. Follow-up ended with the outcome of interest or any of the censoring events, whichever came first. Not having completed secondary school at age 19 was analyzed using conditional logistic regression. Both unadjusted and adjusted analyzes were conducted. The adjusted analyzes included all covariates regarding parental demographics, parental clinical history, parental history of social benefits, and offspring clinical history. Stratified analyzes were also conducted on subsamples based on age of the offspring: 6–10 years or 11–15 years. Offspring to parents without depression was selected as the reference group. Robust standard errors were calculated. Proportional hazards were tested and met using Schoenfeld residuals.

Additional stratified analyzes were also conducted on subsamples based on either the sex of the offspring or the sex of the parent, in combination with age of the offspring.

## Results

We identified 2,359 offspring of parents with TRD, who were matched with 2,358 comparators from the general population and with 2,350 comparators with parents with other depression. 5% (125 of 2350) parents with other depression were later included in the TRD parental cohort. Ten offspring of parents with TRD could only be matched with one comparator each according to the matching criteria.

Table 1 displays the general characteristics of the parent-offspring pair cohorts at baseline. The parents with TRD were more often women (64%) than men (36%) and on average 37 years old at the index AD fill date. The offspring to parents with TRD had a more balanced sex ratio (52% boys and 48% girls) and were on average 10 years old when the parent fulfilled the TRD criteria. As these were matching variables, proportions were similar among the comparators.

**Table 1:** Characteristics of parents with treatment-resistant depression (TRD) and their offspring aged 6–15 years, and of two matched parent-offspring sets: parent-offspring comparators with other parental depression, and parent-offspring comparators from the general population

	Parent with TRD	Parent with non-TRD depression	General population parents	*P* ^1^
***N***	2,359	2,350	2,358	
**Parent mean age**,** years (**± **SD)**	37 (± 6)	37 (± 6)	37 (± 6)	Matched
**Parent sex**				
Women	1,504 (63.8%)	1,503 (64.0%)	1,504 (63.8%)	Matched
Men	855 (36.2%)	847 (36.0%)	854 (36.2%)	
**Parent type of living area** ^2^				
Cities	831 (35.2%)	829 (35.3%)	831 (35.2%)	Matched
Towns and suburbs	1,067 (45.2%)	1,066 (45.4%)	1,067 (45.3%)	
Rural areas	461 (19.5%)	455 (19.4%)	460 (19.5%)	
**Parent country of birth** ^2^				
Sweden	1,825 (77.4%)	1,746 (74.3%)	1,800 (76.3%)	0.014
Europe (other than Sweden)	247 (10.5%)	235 (10.0%)	236 (10.0%)	
Rest of the world	287 (12.2%)	369 (15.7%)	322 (13.7%)	
**Parent educational level** ^2^				
Primary school (≤ 9 years)	394 (16.7%)	375 (16.0%)	290 (12.3%)	< 0.001
Secondary school (10–12 years)	1,223 (51.8%)	1,168 (49.7%)	1,123 (47.6%)	
University/college (> 12 years)	742 (31.5%)	807 (34.3%)	945 (40.1%)	
**Parent family situation** ^2^				
Married or cohabitant without children	11 (0.5%)	11 (0.5%)	6 (0.3%)	< 0.001
Married or cohabitant with children	1,574 (66.7%)	1,460 (62.1%)	1,946 (82.5%)	
Single household without children	278 (11.8%)	354 (15.1%)	138 (5.9%)	
Single household with children	496 (21.0%)	525 (22.3%)	268 (11.4%)	
**Parent number of children** ^2^				
1	562 (23.8%)	613 (26.1%)	454 (19.3%)	< 0.001
2	1,273 (54.0%)	1,236 (52.6%)	1,361 (57.7%)	
≥3	524 (22.2%)	501 (21.3%)	543 (23.0%)	
**Parent history of depression** ^3^				
No	2,303 (97.6%)	2,292 (97.5%)	2,358 (100.0%)	< 0.001^5^
Yes	56 (2.4%)	58 (2.5%)	0 (0.0%)	
**Parent history of substance use disorder** ^3^				
No	2,237 (94.8%)	2,202 (93.7%)	2,348 (99.6%)	< 0.001
Yes, alcohol only	78 (3.3%)	88 (3.7%)	5 (0.2%)	
Yes, other/combined	44 (1.9%)	60 (2.6%)	5 (0.2%)	
**Parent history of other psychiatric disorders** ^3^				
No	2,098 (88.9%)	2,038 (86.7%)	2,344 (99.4%)	< 0.001
Yes	261 (11.1%)	312 (13.3%)	14 (0.6%)	
**Parent history of self-harm/suicide attempt** ^3^				
No	2,311 (98.0%)	2,279 (97.0%)	2,348 (99.6%)	< 0.001
Yes	48 (2.0%)	71 (3.0%)	10 (0.4%)	
**Parent history of non-psychiatric healthcare use** ^3^				
No	709 (30.1%)	738 (31.4%)	1,241 (52.6%)	< 0.001
Yes	1,650 (69.9%)	1,612 (68.6%)	1,117 (47.4%)	
**Parent sick leave days last 365 days**				
0	1,442 (61.1%)	1,528 (65.0%)	2,216 (94.0%)	< 0.001
0–90	698 (29.6%)	598 (25.4%)	114 (4.8%)	
90–365	219 (9.3%)	224 (9.5%)	28 (1.2%)	
**Parent disability pension last 365 days**				
No	2,287 (96.9%)	2,242 (95.4%)	2,341 (99.3%)	< 0.001
Yes	72 (3.1%)	108 (4.6%)	17 (0.7%)	
**Offspring mean age**,** years (**± **SD)**	10 (± 3)	10 (± 3)	10 (± 3)	Matched
**Offspring sex**				
Women	1,135 (48.1%)	1,130 (48.1%)	1,134 (48.1%)	Matched
Men	1,224 (51.9%)	1,220 (51.9%)	1,224 (51.9%)	
**Offspring history of psychiatric diagnosis** ^4^				
No	2,043 (86.6%)	2,060 (87.7%)	2,235 (94.8%)	< 0.001
Yes	316 (13.4%)	290 (12.3%)	123 (5.2%)	
**Offspring history of self-harm** ^4^				
No	2,342 (99.3%)	2,338 (99.5%)	2,337 (99.1%)	0.297
Yes	17 (0.7%)	12 (0.5%)	21 (0.9%)	
**Offspring history of non-psychiatric healthcare use** ^4^				
No	1,839 (78.0%)	1,869 (79.5%)	2,004 (85.0%)	< 0.001
Yes	520 (22.0%)	481 (20.5%)	354 (15.0%)	

^1^ Pearson’s chi-squared test, three-group comparison

^2^At end of calendar year before matching date

^3^≤5 years before index dispensing

^4^At any time before matching date

^5^General population parents have no history of depression by design

SD = Standard deviation

Among parents without depression, 76% were born in Sweden compared to 77% among parents with TRD and 74% of parents without TRD. Parents without depression had more often obtained higher education (40% vs. TRD 32% and other depression 34%). They were also more often married/cohabitant with children living at home (83% vs. 67% and 62%) and had more often > 1 child (81% vs. 76% and 74%). Parents with TRD and parents with other depression were almost equally likely to have a history of depression (around 2%) and a history of substance use disorder (5% vs. 6%), but parents with other depression more often had a history of other psychiatric disorders (13% vs. 11%) and a history of self-harm (3% vs. 2%). History of non-psychiatric healthcare use was more common among parents with TRD and parents with other depression compared to parents without depression (70% and 69% vs. 47%). Parents with TRD were to a higher degree on sick leave in the last 365 days compared to parents with other depression (39% vs. 35%), but parents with other depression had higher rates of disability pension (5% vs. 3%).

Offspring to parents with TRD and offspring to parents with other depression more often had a history of psychiatric disorders than offspring to parents without depression (13% and 12% vs. 5%) as well as non-psychiatric healthcare use (22% and 21% vs. 15%). History of self-harm was not common in any offspring group (< 1%).

In the analysis comparing offspring of parents with TRD with the matched general population offspring, hazards were increased for all outcomes among those with parents who had TRD (Table 2). Their risk for visits to psychiatric services was 3.3 times higher (aHR, 95%CI 2.8-4.0), for depression 4.6 times higher (3.2–6.5), for psychiatric medication 3.6 times higher (3.0–4.2), and for self-harm/suicide attempts 3.2 times higher (1.9–5.5). Among the 714 offspring to parents with TRD who had reached age 19 during the follow-up time, the risk for high-school non- completion was 2.1 times higher (aOR, 1.5–2.9), for sick leave due to mental health reasons 2.3 times higher (aHR 1.1–4.6), and for disability pension 4.2 times higher (aHR 2.2–8.1) than for the general population comparators. The number of suicides was less than 5 in the combined cohorts and this outcome was not further analyzed.

**Table 2:** Survival analysis. Outcomes for matched offspring of parents with treatment resistant depression (TRD), non-TRD depression, and no depression (reference). Incidence rates (IR) per 100,000 person years and crude and adjusted hazard ratios (HR, aHR) and odds ratios (OR, aOR) with 95% confidence intervals (CI)

		Offspring of parent with TRD						Offspring of parent with non-TRD depression						Offspring of general population parent		
Age at matching date	**Total N**	**N**	**IR**	**HR**	**95%CI**	**aHR** ^**1**^	**95%CI**	**N**	**IR**	**HR**	**95%CI**	**aHR** ^**1**^	**95%CI**	**N**	**IR**	**HR**
**Overall contact with psychiatry**																
All	7067	641	5235	4.2	(3.6–4.8)	3.3	(2.8-4.0)	607	5263	4.1	(3.5–4.8)	3.3	(2.8–3.9)	182	1306	Ref.
6–10	3669	283	4444	4.2	(3.4–5.3)	3.7	(2.9–4.8)	261	4306	4.0	(3.2-5.0)	3.5	(2.7–4.5)	78	1101	Ref.
11–15	3398	358	6094	4.1	(3.4-5.0)	3.2	(2.5-4.0)	346	6323	4.2	(3.4–5.1)	3.3	(2.7–4.1)	104	1518	Ref.
**Depression diagnosis**																
All	7067	177	1237	4.7	(3.4–6.4)	4.6	(3.2–6.5)	164	1217	4.6	(3.4–6.3)	4.4	(3.1–6.2)	40	275	Ref.
6–10	3669	55	748	6.3	(3.4–12.0)	9.0	(4.2–19.4)	51	735	6.4	(3.3–12.4)	10.1	(4.5–22.5)	9	122	Ref.
11–15	3398	122	1752	4.2	(2.9-6.0)	3.9	(2.6–5.8)	113	1727	4.1	(2.9–5.9)	3.7	(2.5–5.5)	31	429	Ref.
**Psychiatric medication**																
All	7067	716	5923	4.2	(3.6–4.8)	3.6	(3.0-4.2)	634	5450	3.7	(3.2–4.3)	3.2	(2.8–3.8)	215	1546	Ref.
6–10	3669	296	4610	4.6	(3.6–5.8)	4.1	(3.2–5.3)	263	4314	4.2	(3.3–5.2)	3.8	(2.9-5.0)	79	1121	Ref.
11–15	3398	420	7411	3.9	(3.3–4.7)	3.4	(2.7–4.1)	371	6702	3.4	(2.9–4.1)	3.0	(2.4–3.7)	136	1984	Ref.
**Suicide attempt**																
All	7067	61	417	3.0	(1.9–4.5)	3.2	(1.9–5.5)	58	420	3.0	(1.9–4.6)	3.3	(1.9–5.6)	21	144	Ref.
6–10	3669	27	365	2.4	(1.3–4.4)	2.7	(1.2–6.1)	21	300	2.0	(1.1–3.9)	1.8	(0.8-4.0)	11	150	Ref.
11–15	3398	34	470	3.5	(1.9–6.5)	3.9	(1.8–8.8)	37	544	4.1	(2.2–7.4)	5.4	(2.4–12.2)	10	138	Ref.
**Sick leave with psychiatric diagnosis** ^2^																
All	2090	40	1850	2.5	(1.5–4.2)	2.3	(1.1–4.6)	30	1466	2.2	(1.3–3.7)	2.5	(1.1–5.6)	16	732	Ref.
6–10	363	2	1168	-	-	-	-	3	1510	-	-	-	-	0	0	Ref.
11–15	1727	38	1909	2.4	(1.5-4.0)	2.1	(1.1–4.3)	27	1461	2.0	(1.2–3.4)	2.2	(1.0-4.9)	16	790	Ref.
**Disability pension for any reason** ^2^																
All	2090	61	2945	3.2	(2.1–4.9)	4.2	(2.2–8.1)	54	2792	2.9	(1.9–4.6)	3.0	(1.7–5.3)	19	888	Ref.
6–10	363	10	6284	4.5	-	-	-	13	7373	-	-	-	-	2	1243	Ref.
11–15	1727	51	2667	3.01	(1.9–4.8)	4.1	(1.9–8.7)	41	2333	2.7	(1.7–4.4)	2.8	(1.5–5.1)	17	859	Ref.
**Age at matching date**	Total N	**N**		**OR**	**95%CI**	**aOR** ^**2**^	**95%CI**	**N**		**OR**	**95%CI**	**aOR** ^**2**^	**95%CI**	**N**		**OR**
**Not completed secondary school at age 19** ^3^																
All	2089	259		2.6	(2.0-3.2)	2.1	(1.5–2.9)	240		2.5	(2.0-3.3)	1.9	(1.3–2.6)	133		Ref.
6–10	363	41		3.1	(1.6–6.1)	3.7	(1.0-13.5)	41		2.3	(1.1–4.9)	2.0	(0.5–7.8)	19		Ref.
11–15	1726	218		2.5	(1.9–3.3)	2.0	(1.4–2.9)	199		2.6	(2.0-3.4)	1.9	(1.3–2.7)	114		Ref.

^1^Adjusted for parental history of substance use disorders, depression, other psychiatric disorders, self-harm/suicide attempt, non-psychiatric health care utilization, disability pension, sickness absence, family situation, number of children and country of birth, as well as offspring history of self-harm/suicide attempt, psychiatric diagnoses and non-psychiatric health care utilization. ^2^Only offspring for whom data is available from July 1 in the calendar year in which they reach 19 years of age. ^3^ Only offspring for whom data is available for the full calendar year in which they reach 19 years of age

Offspring of parents with other depression showed similar results for all outcomes when compared to the general population offspring. A direct comparison between the offspring of parents with TRD vs. other depression regarding all outcomes showed non-significant differences with point estimates ranging from 0.7 to 1.1 (Table 3).

**Table 3:** Survival analysis. Outcomes for matched offspring of parents with treatment resistant depression (TRD) vs. non-TRD depression. Incidence rates (IR) per 100,000 person years and crude and adjusted hazard ratios (HR, aHR) and odds ratios (OR, aOR) with 95% confidence intervals (CI)

Offspring age in years at start of follow-up	Offspring of parent with TRD						Offspring of parent with non-TRD depression		
	*N*	IR	HR	95%CI	aHR^1^	95%CI	*N*	IR	HR
**Overall contact with psychiatry**									
All	641	5235	1.0	(0.9–1.1)	1.0	(0.9–1.1)	607	5263	Ref.
6–10	283	4444	1.1	(0.9–1.2)	1.1	(0.9–1.2)	261	4306	Ref.
11–15	358	6094	1.0	(0.9–1.1)	1.0	(0.8–1.1)	346	6323	Ref.
**Depression diagnosis**									
All	177	1237	1.0	(0.9–1.2)	1.0	(0.9–1.3)	164	1217	Ref.
6–10	55	748	1.0	(0.7–1.3)	0.9	(0.6–1.3)	51	735	Ref.
11–15	122	1752	1.0	(0.8–1.2)	1.1	(0.8–1.3)	113	1727	Ref.
**Psychiatric medication**									
All	716	5923	1.1	(1.0-1.2)	1.1	(1.0-1.2)	634	5450	Ref.
6–10	296	4610	1.1	(1.0-1.3)	1.1	(0.9–1.3)	263	4314	Ref.
11–15	420	7411	1.2	(1.0-1.3)	1.1	(1.0-1.3)	371	6702	Ref.
**Suicide attempt**									
All	61	417	1.0	(0.8–1.3)	1.0	(0.7–1.4)	58	420	Ref.
6–10	27	365	1.2	(0.8–1.9)	1.6	(0.8–2.9)	21	300	Ref.
11–15	34	470	0.9	(0.6–1.3)	0.7	(0.5–1.2)	37	544	Ref.
**Sick leave with psychiatric diagnosis** ^2^									
All	40	1850	1.2	(0.8–1.7)	0.9	(0.5–1.6)	30	1466	Ref.
6–10	2	1168	-	-	-	-	3	1510	Ref.
11–15	38	1909	1.2	(0.8–1.8)	1.0	(0.6–1.6)	27	1461	Ref.
**Disability pension with any diagnosis** ^2^									
All	61	2945	1.1	(0.8–1.5)	1.4	(0.9–2.2)	54	2792	Ref.
6–10	10	6284	-	-	-	-	13	7373	Ref.
11–15	51	2667	1.1	(0.8–1.6)	1.5	(0.9–2.5)	41	2333	Ref.
	**N**		**OR**	**95%CI**	**aOR** ^**2**^	**95%CI**	**N**		**OR**
**Not completed secondary school at age 19** ^3^									
All	259		1.0	(0.8–1.3)	1.1	(0.9–1.5)	133		Ref.
6–10	51		1.4	(0.8–2.5)	1.8	(0.8–4.1)	19		Ref.
11–15	218		1.0	(0.8–1.3)	1.1	(0.8–1.3)	114		Ref.

^1^Adjusted for parental history of substance use disorders, depression, other psychiatric disorders, self-harm/suicide attempt, non-psychiatric health care utilization, disability pension, sickness absence, family situation, number of children and country of birth, as well as offspring history of self-harm/suicide attempt, psychiatric diagnoses and non-psychiatric health care utilization. ^2^Only offspring for whom data is available from July 1 in the calendar year in which they reach 19 years of age. ^3^ Only offspring for whom data is available for the full calendar year in which they reach 19 years of age

Results stratified by age were generally similar to results in the main analysis, and any differences in relative risks were nonsignificant with overlapping CIs. Likewise, in analyses stratified by parental and offspring sex, the results were overall similar although with wider CIs; results for the outcomes of sick leave, disability pension and secondary school completion were not interpretable for the age group of 6–10 due to small cell numbers. Overall, no significant effect modifications could be found (Suppl Tables 2 to 5).

## Discussion

Results in the present study show that offspring of parents with TRD and other depression had substantially higher risks for various negative health, education and work disability outcomes compared to matched offspring of parents from the general population. However, contrary to our hypothesis, parental TRD was not associated with any further increase in this risk for the offspring compared to other parental depression.

While TRD is originally a clinically derived concept, pharmacoepidemiological definitions of TRD have been extensively used in epidemiological research over the past decade [24–27, 36]. Such studies have consistently reported an elevated risk of negative outcomes, including mortality, psychiatric hospitalization, work disability, and substance abuse in TRD compared to other patients with depression [24, 28, 29, 37]. However, this elevated risk did not transfer to their offspring in the present study. This may be due to our conservative study design, limiting the inclusion of patients with depression to those in specialized psychiatric care receiving pharmacological treatment. This means that patients with TRD and other depression alike had substantial morbidity at baseline, which is likely to have reduced the risk differences compared to if parents with depression identified and treated in primary care had been included. The fact that parents with other depression had higher rates of psychiatric morbidity at baseline compared to those with TRD in this study suggests a delineation of healthier patients being selected for receiving optimization of treatment for depression. This bias may have implications for the interpretation of future epidemiological research on TRD.

The label TRD is generally applied to a single depressive episode, and in the present study we restricted the measurement time for TRD to one year to avoid measuring treatment of recurrent episodes. This time period may, however, not sufficiently capture a time spent in a depressive state long enough to impact offspring outcomes. The *chronicity* of parental depression has been linked in clinical and database cohorts to increased risks for negative outcomes in offspring, e.g. depression, suicidality, and externalizing behavior - often independent of the severity of the parental depressive episode [38–41]. Two other potentially influential factors that may occur outside the one-year timeframe in the present study are spontaneous remission of depression, which occurs in 24% of patients around 12 months into the episode [42], and, conversely, the recurrence of depression after initial remission, affecting 25% of patients within 1–3 years after remission and 40–60% of patients during their lifetime [43, 44]. These factors could potentially attenuate our results.

In addition to chronicity, *non-remission* in depression is closely related to, but not identical with, TRD. A systematic review of 10 studies investigated the impact of parental depression improvement on psychopathological and developmental outcomes in the offspring [45]. Most of the included studies reported associations between parental improvement and lower risk of, or improvement in, negative offspring outcomes. Further, in a study comparing offspring of mothers with and without remission 3 months into treatment of a depressive episode in the STAR*D trial [46], remission was associated with reductions in offspring psychiatric diagnoses and symptoms. Another study by the same author found that maternal depression relapse increased the psychiatric symptom burden in the offspring [47]. Similarly, a study following mother-offspring pair longitudinally showed that parental depression scores exhibited curvilinear trajectories - initial decrease followed by increase - over two years, with offspring depression scores following the same pattern [20]. These findings of parental remission being protective against negative offspring outcomes are however not directly comparable to the concept of TRD, in which we also had an extended follow-up time focused on real-life outcomes rather than symptom scores.

Strengths of this study include the population-based setting using nationwide registers of high validity [30] and almost total population coverage, allowing for the identification of a comparatively large cohort of parents with TRD, along with sociodemographic and clinical covariates and outcomes. Using objectively measured clinical and sociodemographic outcomes minimizes the risk for subjective and rater bias compared to e.g. depression rating scales and identifies relevant clinical and functional detrimental outcomes. A limitation of the reported outcomes is that mental problems not leading to contacts with health care or having an impact on educational results or work disability are not captured. Parents with depression may have a higher threshold for identifying and seeking health care for mental health care needs in their offspring. On the other hand, needs of offspring to parents with a psychiatric contact may be more readily identified by health care and social services, creating uncertainty of the direction of a possible detection bias. Another limitation of the study is the algorithm-based definition of TRD, lacking clinical data such as rating scales or reasons for antidepressant treatment discontinuation. In order to investigate different aspects of clinical and social functioning we did not take correlation between the separate outcomes – though likely considerable - into account, which does not allow for investigating mediating effects between the outcomes, e.g. to what extent the effects on school and work ability were due to depression in the offspring. The correlation between individuals and their offspring among patients with TRD compared with patients with other depression was not investigated. As groups were slightly overlapping, this may warrant further study.

Furthermore, our study focused on the effect of treatment resistance in a single episode and did not consider recurrence or temporal trajectories. Additionally, the follow-up did not allow for sufficient statistical power in measuring all outcomes, specifically work disability outcomes in the youngest offspring as it was measured only among those who reached age 18 during the study follow-up.

In conclusion, our long-term follow-up study showed that offspring of parents with depression have a markedly higher risk of adverse psychiatric, educational and work-related outcomes compared to children of parents in the general population. A treatment-resistant depressive episode in the parent does not appear to further increase the risk for clinical and work-related outcomes in offspring of school age compared to non-treatment resistant depression.

## Supplementary Information

Below is the link to the electronic supplementary material.

Supplementary Material 1

## Data Availability

Data is available by order from the relevant Swedish authorities upon formal request and ethical permission.
