# Supplementary material for: Psychiatric outcomes and long-term school and work-related disability in offspring of parents with depression and treatment-resistant depression
Source: Soc Psychiatry Psychiatr Epidemiol. 2025 Sep 22;61(3):423–34. doi: 10.1007/s00127-025-02988-z (PMC12996023; doi:10.1007/s00127-025-02988-z)
Supplement: Supplementary file 1 — Supplementary Material 1 [file 127_2025_2988_MOESM1_ESM.docx]

**Supplemental Table 1. International Classification of Diseases, 10^th^ edition (ICD-10) codes and Anatomical Therapeutic Chemical (ATC) Classification codes for variables applied in the study.**

|  | ICD-10 | ATC |
| --- | --- | --- |
| Dementia | F00-03, G30 |  |
| Psychotic disorders | F20-29 |  |
| Manic episode | F30 |  |
| Bipolar disorder | F31 |  |
| History of depression | F32-F33 |  |
| Anxiety disorders | F40-F41 |  |
| Obsessive-compulsive disorder | F42 |  |
| Eating disorders | F50 |  |
| Personality disorders | F60-61 |  |
| Autism spectrum disorder | F84 0-1, F84.5 |  |
| Attention deficit and hyperactivity disorders | F90 |  |
| Alcohol use disorder only | F10.1-9 | and/or disulfiram (N07BB01) acamprosate (N07BB03) naltrexone (N07BB04) nalmefen (N07BB05) |
| Other/combined substance use disorder | F11-16 and/or F18-19, alone or in combination with F10.1-9 | and/or sublingual buprenorphine (N07BC01/ N07BC51), methadone (N07BC02), naloxone (V03AB15) – alone or in combination with ATC codes for alcohol use disorder |
| Electroconvulsive therapy | DA006, DA024-5 |  |
| Vagus nerve stimulation | DA022 |  |
| Repetitive transcranial magnetic stimulation | DU050 |  |
| Self harm/suicide attempt | X60-84, Y10-34 |  |
| Antidepressants |  | N06A |
| Mood stabililizers |  | carbamazepine (N03AF01), valproate (N03AG01), lamotrigine (N03AX09) |
|  |  |  |
| Antipsychotics and lithium |  | N05A |
| Non-psychiatric health care visit | A00-E90 and G00-N99, as main diagnoses only |  |
| Dispensed psychiatric medication |  | N05A-B, N05C, N06A-B |

**Supplemental table 2.** **Female offspring only.**

| Outcomes for matched offspring of parents with treatment resistant depression (TRD), other depression, and no depression (Ref.erence). Incident rates (IR) per 100,000 person years and crude and adjusted hazard ratios (HR, aHR) and odds ratios (OR, aOR) with 95% confidence intervals (CI). | | **Offspring of parent with TRD** | | | | | | **Offspring of parent with other depression** | | | | | | **Offspring of general**  **population parent** | | | |
| --- | --- | --- | --- | --- | --- | --- | --- | --- | --- | --- | --- | --- | --- | --- | --- | --- | --- |
| **Age at start of follow-up** | **Total N** | **N** | **IR** | **HR** | **95%CI** | **aHR^1^** | **95%CI** | **N** | **IR** | **HR** | **95%CI** | **aHR^1^** | **95%CI** | **N** | **IR** | **HR** |  |
| **Overall contact with psychiatry** | |  |  |  |  |  |  |  |  |  |  |  |  |  |  |  | |
| All | 3399 | 306 | 5151 | 4.0 | (3.2-4.9) | 3.7 | (2.9-4.7) | 282 | 4919 | 3.8 | (3.1-4.7) | 3.4 | (2.7-4.3) | 91 | 1356 | Ref. |  |
| 6-10 | 1772 | 132 | 4176 | 4.2 | (3.0-5.9) | 4.1 | (2.8-6.1) | 108 | 3556 | 3.5 | (2.5-4.9) | 3.3 | (2.2-4.8) | 34 | 981 | Ref. |  |
| 11-15 | 1627 | 174 | 6259 | 3.8 | (2.9-5.0) | 3.5 | (2.6-4.7) | 174 | 6453 | 4.0 | (3.1-5.3) | 3.7 | (2.7-5.0) | 57 | 1758 | Ref. |  |
| **Depression diagnosis** | |  |  |  |  |  |  |  |  |  |  |  |  |  |  |  | |
| All | 3399 | 107 | 1569 | 3.5 | (2.5-5.0) | 3.5 | (2.4-5.2) | 109 | 1700 | 4.0 | (2.8-5.6) | 3.8 | (2.6-5.7) | 32 | 459 | Ref. |  |
| 6-10 | 1772 | 34 | 956 | 4.5 | (2.3-8.8) | 7.8 | (2.9-20.8) | 35 | 1055 | 5.0 | (2.5-10.1) | 8.9 | (3.1-25.3) | 8 | 224 | Ref. |  |
| 11-15 | 1627 | 73 | 2238 | 3.2 | (2.1-4.8) | 3.1 | (2.0-4.9) | 74 | 2392 | 3.6 | (2.4-5.4) | 3.4 | (2.2-5.4) | 24 | 706 | Ref. |  |
| **Psychiatric medication** | |  |  |  |  |  |  |  |  |  |  |  |  |  |  |  | |
| All | 3399 | 354 | 6035 | 3.7 | (3.1-4.5) | 3.6 | (2.9-4.5) | 309 | 5419 | 3.1 | (2.6-3.8) | 3.1 | (2.5-3.9) | 120 | 1802 | Ref. |  |
| 6-10 | 1772 | 149 | 4699 | 4.8 | (3.4-6.8) | 5.2 | (3.4-7.8) | 112 | 3687 | 3.5 | (2.5-4.9) | 3.7 | (2.4-5.6) | 37 | 1074 | Ref. |  |
| 11-15 | 1627 | 205 | 7605 | 3.2 | (2.6-4.0) | 3.0 | (2.3-4.0) | 197 | 7394 | 2.9 | (2.4-3.7) | 3.0 | (2.3-4.0) | 83 | 2582 | Ref. |  |
| **Suicide attempt** | |  |  |  |  |  |  |  |  |  |  |  |  |  |  |  | |
| All | 3399 | 37 | 527 | 3.1 | (1.8-5.4) | 3.7 | (1.8-7.4) | 35 | 528 | 3.2 | (1.8-5.7) | 3.3 | (1.6-6.7) | 12 | 171 | Ref. |  |
| 6-10 | 1772 | 18 | 501 | 2.9 | (1.3-6.5) | 5.0 | 1.0-26.0) | 14 | 417 | 2.5 | (1.1-5.8) | 3.4 | (0.6-18.0) | 6 | 169 | Ref. |  |
| 11-15 | 1627 | 19 | 554 | 3.3 | (1.5-7.1) | 3.7 | (1.1-12.7) | 21 | 642 | 4.0 | (1.8-8.6) | 5.0 | (1.5-17.0) | 6 | 174 | Ref. |  |
| **Sick leave with psychiatric diagnosis**^2^ | |  |  |  |  |  |  |  |  |  |  |  |  |  |  |  | |
| 11-15 | 809 | 21 | 2292 | 1.5 | (0.9-2.7) | 1.03 | (0.4-2.5) | 20 | 2276 | 1.6 | (0.9-2.9) | 1.2 | (0.5-2.9) | 14 | 1503 | Ref. |  |
| **Disability pension for any reason**^2^ | |  |  |  |  |  |  |  |  |  |  |  |  |  |  |  | |
| 11-15 | 809 | 21 | 2361 | 7.0 | (2.4-20.6) | N/A |  | 18 | 2114 | 7.1 | (2.5-20.2) | N/A |  | 3 | 319 | Ref. |  |
| **Age at start of follow-up** | **total N** | **N** |  | **OR** | **95%CI** | **aOR^2^** | **95%CI** | **N** |  | **OR** | **95%CI** | **aOR^2^** | **95%CI** | **N** |  | **OR** |  |
| **Not completed secondary school in year when aged 19**^3^ | |  |  |  |  |  |  |  |  |  |  |  |  |  |  |  | |
| 11-15 | 808 | 85 |  | 2.5 | (1.6-3.8) | 2.0 | (1.2-3.4) | 86 |  | 2.7 | (1.8-4.2) | 2.0 | (1.2-3.6) | 43 |  | Ref. |  |

^1^Adjusted for parental history of substance use disorders, depression, other psychiatric disorders, self-harm/suicide attempt, non-psychiatric health care utilization, disability pension, sickness absence, family situation, number of children and country of birth, as well as offspring history of self-harm/suicide attempt, psychiatric diagnoses and non-psychiatric health care utilization. ^2^Only offspring for whom data is available from July 1 in the calendar year in which they reach 19 years of age. ^3^ Only offspring for whom data is available for the full calendar year in which they reach 19 years of age.

**Supplemental table 3.** **Male offspring only.** Outcomes for matched offspring of parents with treatment resistant depression (TRD), other depression, and no depression (Ref.erence). Incident rates (IR) per 100,000 person years and crude and adjusted hazard ratios (HR, aHR) and odds ratios (OR, aOR) with 95% confidence intervals (CI).

|  | | **Offspring of parent with TRD** | | | | | | **Offspring of parent with other depression** | | | | | | **Offspring of general**  **population parent** | | | |
| --- | --- | --- | --- | --- | --- | --- | --- | --- | --- | --- | --- | --- | --- | --- | --- | --- | --- |
| **Age at start of follow-up** | **total N** | **N** | **IR** | **HR** | **95%CI** | **aHR^1^** | **95%CI** | **N** | **IR** | **HR** | **95%CI** | **aHR^1^** | **95%CI** | **N** | **IR** | **HR** |  |
| **Overall contact with psychiatry** | |  |  |  |  |  |  |  |  |  |  |  |  |  |  |  | |
| All | 3668 | 335 | 5315 | 4.3 | (3.5-5.4) | 3.1 | (2.4-3.9) | 325 | 5604 | 4.4 | (3.6-5.5) | 3.3 | (2.6-4.1) | 91 | 1259 | Ref. |  |
| 6-10 | 1897 | 151 | 4707 | 4.2 | (3.1-5.8) | 3.7 | (2.6-5.2) | 153 | 5060 | 4.5 | (3.3-6.1) | 4.0 | (2.8-5.6) | 44 | 1216 | Ref. |  |
| 11-15 | 1771 | 184 | 5945 | 4.4 | (3.3-6.0) | 2.8 | (2.0-4.0) | 172 | 6197 | 4.4 | (3.3-5.8) | 3.0 | (2.2-4.3) | 47 | 1302 | Ref. |  |
| **Depression diagnosis** | |  |  |  |  |  |  |  |  |  |  |  |  |  |  |  | |
| All | 3668 | 70 | 934 | 9.3 | (4.7-18.3) | 9.4 | (4.1-21.0) | 55 | 778 | 7.3 | (3.7-14.6) | 7.6 | (3.5-16.9) | 8 | 105 | Ref. |  |
| 6-10 | 1897 | 21 | 554 | 21.0 | (3.0-149.0) | 1726 | (39.4-75609) | 16 | 442 | 17.6 | (2.4-128) | 119.7 | (9.2-1553) | 1 | 26 | Ref. |  |
| 11-15 | 1771 | 49 | 1323 | 7.6 | (3.7-15.7) | 6.6 | (2.8-15.4) | 39 | 1131 | 5.9 | (2.8-12.2) | 5.5 | (2.4-12.6) | 7 | 183 | Ref. |  |
| **Psychiatric medication** | |  |  |  |  |  |  |  |  |  |  |  |  |  |  |  | |
| All | 3668 | 362 | 5818 | 4.7 | (3.8-5.8) | 3.7 | (2.9-4.7) | 325 | 5480 | 4.5 | (3.6-5.5) | 3.6 | (2.8-4.6) | 95 | 1311 | Ref. |  |
| 6-10 | 1897 | 147 | 4523 | 4.3 | (3.2-6.0) | 3.6 | (2.5-5.1) | 151 | 4936 | 4.8 | (3.5-6.6) | 4.1 | (2.8-5.9) | 42 | 1165 | Ref. |  |
| 11-15 | 1771 | 215 | 7234 | 5.1 | (3.8-6.7) | 4.0 | (2.9-5.7) | 174 | 6060 | 4.2 | (3.1-5.6) | 3.5 | (2.4-4.9) | 53 | 1455 | Ref. |  |
| **Suicide attempt** | |  |  |  |  |  |  |  |  |  |  |  |  |  |  |  | |
| All | 3668 | 24 | 315 | 2.8 | (1.4-5.3) | 3.3 | (1.3-8.9) | 23 | 321 | 2.7 | (1.4-5.3) | 4.0 | (1.5-10.7) | 9 | 119 | Ref. |  |
| 6-10 | 1897 | 9 | 237 | 1.8 | (0.7-4.5) | N/A |  | 7 | 193 | 1.5 | (0.6-4.1) | N/A |  | 5 | 133 | Ref. |  |
| 11-15 | 1771 | 15 | 394 | 3.9 | (1.5-10.1) | 3.1 | (0.2-42.1) | 16 | 452 | 4.2 | (1.7-10.8) | 5.5 | (1.0-30.8) | 4 | 105 | Ref. |  |
| **Sick leave with psychiatric diagnosis**^2^ | |  |  |  |  |  |  |  |  |  |  |  |  |  |  |  | |
| 11-15 | 918 | 17 | 1582 | 8.5 | (2.2-34.0) |  |  | 7 | 722 | 4.4 | 1.0-20.1) |  |  | 2 | 183 | Ref. |  |
| **Disability pension for any reason**^2^ | |  |  |  |  |  |  |  |  |  |  |  |  |  |  |  | |
| 11-15 | 918 | 30 | 2934 | 2.1 | (1.3-3.6) | 3.5 | (1.4-8.4) | 23 | 2538 | 1.8 | (1.1-3.2) | 1.9 | (0.8-4.6) | 14 | 1346 | Ref. |  |
| **Age at start of follow-up** | **total N** | **N** |  | **OR** | **95%CI** | **aOR^2^** | **95%CI** | **N** |  | **OR** | **95%CI** | **aOR^2^** | **95%CI** | **N** |  | **OR** |  |
| **Not completed secondary school in year when aged 19**^3^ | |  |  |  |  |  |  |  |  |  |  |  |  |  |  |  | |
| 11-15 | 918 | 133 |  | 2.5 | (1.8-3.6) | 2.1 | (1.3-3.4) | 113 |  | 2.5 | (1.7-3.6) | 1.8 | (1.1-3.0) | 71 |  | Ref. |  |

^1^Adjusted for parental history of substance use disorders, depression, other psychiatric disorders, self-harm/suicide attempt, non-psychiatric health care utilization, disability pension, sickness absence, family situation, number of children and country of birth, as well as offspring history of self-harm/suicide attempt, psychiatric diagnoses and non-psychiatric health care utilization. ^2^Only offspring for whom data is available from July 1 in the calendar year in which they reach 19 years of age. ^3^ Only offspring for whom data is available for the full calendar year in which they reach 19 years of age.

**Supplemental table 4.** **Only offspring linked with female parents.** Outcomes for matched offspring of parents with treatment resistant depression (TRD), other depression, and no depression (Ref.erence). Incident rates (IR) per 100,000 person years and crude and adjusted hazard ratios (HR, aHR) and odds ratios (OR, aOR) with 95% confidence intervals (CI).

|  | | **Offspring of parent with TRD** | | | | | | **Offspring of parent with other depression** | | | | | | **Offspring of general**  **Population parent** | | | |
| --- | --- | --- | --- | --- | --- | --- | --- | --- | --- | --- | --- | --- | --- | --- | --- | --- | --- |
| **Age at start of follow-up** | **Total N** | **N** | **IR** | **HR** | **95%CI** | **aHR^1^** | **95%CI** | **N** | **IR** | **HR** | **95%CI** | **aHR^1^** | **95%CI** | **N** | **IR** | **HR** |  |
| **Overall contact with psychiatry** | |  |  |  |  |  |  |  |  |  |  |  |  |  |  |  | |
| All | 4511 | 409 | 5144 | 4.0 | (3.3-4.8) | 3.4 | (2.8-4.1) | 407 | 5494 | 4.0 | (3.4-4.9) | 3.5 | (2.9-4.2) | 122 | 1360 | Ref. |  |
| 6-10 | 2367 | 182 | 4345 | 4.3 | (3.2-5.7) | 4.4 | (3.1-6.1) | 179 | 4554 | 4.3 | (3.3-5.7) | 4.3 | (3.1-6.0) | 50 | 1081 | Ref. |  |
| 11-15 | 2144 | 227 | 6034 | 3.7 | (2.9-4.7) | 2.9 | (2.2-3.8) | 228 | 6557 | 3.9 | (3.0-4.9) | 3.1 | (2.4-4.0) | 72 | 1657 | Ref. |  |
| **Depression diagnosis** | |  |  |  |  |  |  |  |  |  |  |  |  |  |  |  | |
| All | 4511 | 123 | 1330 | 4.5 | (3.1-6.5) | 4.7 | (3.0-7.1) | 114 | 1305 | 4.5 | (3.1-6.6) | 4.6 | (3.0-7.0) | 29 | 308 | Ref. |  |
| 6-10 | 2367 | 37 | 768 | 7.5 | (3.1-18.2) | 14.0 | (4.6-43.1) | 37 | 813 | 8.3 | (3.4-20.3) | 16.6 | (5.1-54.0) | 5 | 104 | Ref. |  |
| 11-15 | 2144 | 86 | 1942 | 3.8 | (2.6-5.8) | 3.6 | (2.2-5.7) | 77 | 1840 | 3.7 | (2.5-5.6) | 3.4 | (2.1-5.5) | 24 | 520 | Ref. |  |
| **Psychiatric medication** | |  |  |  |  |  |  |  |  |  |  |  |  |  |  |  | |
| All | 4511 | 477 | 6121 | 4.0 | (3.3-4.7) | 3.6 | (3.0-4.3) | 426 | 5704 | 3.6 | (3.0-4.2) | 3.3 | (2.7-4.0) | 149 | 1664 | Ref. |  |
| 6-10 | 2367 | 200 | 4768 | 4.4 | (3.3-5.9) | 4.6 | (3.3-6.3) | 183 | 4632 | 4.2 | (3.2-5.5) | 4.3 | (3.1-6.0) | 54 | 1174 | Ref. |  |
| 11-15 | 2144 | 277 | 7699 | 3.7 | (3.0-4.6) | 3.2 | (2.5-4.0) | 243 | 6908 | 3.2 | (2.6-4.0) | 2.9 | (2.2-3.7) | 95 | 2183 | Ref. |  |
| **Suicide attempt** | |  |  |  |  |  |  |  |  |  |  |  |  |  |  |  | |
| All | 4511 | 40 | 422 | 2.9 | (1.7-4.9) | 3.4 | (1.8-6.5) | 44 | 493 | 3.4 | (2.0-5.7) | 4.6 | (2.3-8.9) | 14 | 148 | Ref. |  |
| 6-10 | 2367 | 15 | 308 | 1.8 | (0.9-3.8) | 3.0 | (1.3-7.0) | 16 | 348 | 2.0 | (1.0-4.2) | 2.5 | (0.7-8.1) | 8 | 168 | Ref. |  |
| 11-15 | 2144 | 25 | 542 | 4.4 | (2.1-9.3) | 6.3 | (2.5-15.4) | 28 | 646 | 5.1 | (2.4-11.0) | 10.3 | (3.6-30.0) | 6 | 128 | Ref. |  |
| **Sick leave with psychiatric diagnosis**^2^ | |  |  |  |  |  |  |  |  |  |  |  |  |  |  |  | |
| 11-15 | 1116 | 19 | 1470 | 1.6 | (0.9-3.0) | 0.8 | (0.3-1.8) | 19 | 1624 | 1.9 | (1.0-3.6) | 3.2 | (1.2-8.9) | 12 | 924 | Ref. |  |
| **Disability pension for any reason**^2^ | |  |  |  |  |  |  |  |  |  |  |  |  |  |  |  | |
| 11-15 | 1116 | 31 | 2511 | 2.8 | (1.6-5.0) | 6.2 | (1.9-20.2) | 26 | 2328 | 2.8 | (1.6-4.8) | 4.1 | (1.9-8.6) | 11 | 864 | Ref. |  |
| **Age at start of follow-up** | **total N** | **N** | **IR** | **OR** | **95%CI** | **aOR^2^** | **95%CI** | **N** | **IR** | **OR** | **95%CI** | **aOR^2^** | **95%CI** | **N** | **IR** | **OR** |  |
| **Not completed secondary school in year when aged 19**^3^ | |  |  |  |  |  |  |  |  |  |  |  |  |  |  |  | |
| 11-15 | 1116 | 145 |  | 2.7 | (1.9-3.8) | 2.3 | (1.5-3.5) | 132 |  | 2.7 | (1.9-3.8) | 2.1 | (1.3-3.3) | 72 |  | Ref. |  |

^1^Adjusted for parental history of substance use disorders, depression, other psychiatric disorders, self-harm/suicide attempt, non-psychiatric health care utilization, disability pension, sickness absence, family situation, number of children and country of birth, as well as offspring history of self-harm/suicide attempt, psychiatric diagnoses and non-psychiatric health care utilization. ^2^Only offspring for whom data is available from July 1 in the calendar year in which they reach 19 years of age. ^3^ Only offspring for whom data is available for the full calendar year in which they reach 19 years of age.

**Supplemental table 5.** **Only offspring linked with male parents.** Outcomes for matched offspring of parents with treatment resistant depression (TRD), other depression, and no depression (Ref.erence). Incident rates (IR) per 100,000 person years and crude and adjusted hazard ratios (HR, aHR) and odds ratios (OR, aOR) with 95% confidence intervals (CI).

|  | | **Offspring of parent with TRD** | | | | | | **Offspring of parent with other depression** | | | | | | **Offspring of general**  **population parent** | | | |
| --- | --- | --- | --- | --- | --- | --- | --- | --- | --- | --- | --- | --- | --- | --- | --- | --- | --- |
| **Age at start of follow-up** | **Total N** | **N** | **IR** | **HR** | **95%CI** | **aHR^1^** | **95%CI** | **N** | **IR** | **HR** | **95%CI** | **aHR^1^** | **95%CI** | **N** | **IR** | **HR** |  |
| **Overall contact with psychiatry** | |  |  |  |  |  |  |  |  |  |  |  |  |  |  |  | |
| All | 2556 | 232 | 5405 | 4.6 | (3.5-5.9) | 3.3 | (2.5-4.5) | 200 | 4849 | 4.2 | (3.2-5.5) | 3.0 | (2.3-4.1) | 60 | 1208 | Ref. |  |
| 6-10 | 1302 | 101 | 4634 | 4.1 | (2.8-6.0) | 3.0 | (1.9-4.6) | 82 | 3849 | 3.5 | (2.4-5.1) | 2.6 | (1.7-4.0) | 28 | 1138 | Ref. |  |
| 11-15 | 1254 | 131 | 6200 | 5.0 | (3.5-7.2) | 4.0 | (2.6-6.3) | 118 | 5916 | 4.9 | (3.4-7.0) | 3.9 | (2.5-6.1) | 32 | 1277 | Ref. |  |
| **Depression diagnosis** | |  |  |  |  |  |  |  |  |  |  |  |  |  |  |  | |
| All | 2556 | 54 | 1066 | 5.2 | (2.9-9.3) | 7.4 | (3.6-15.1) | 50 | 1054 | 4.9 | (2.7-8.8) | 5.7 | (2.8-11.3) | 11 | 213 | Ref. |  |
| 6-10 | 1302 | 18 | 712 | 5.0 | (2.11-11.9) | 5.5 | (1.8-16.8) | 14 | 587 | 4.0 | (1.5-10.6) | 5.4 | (1.7-17.0) | 4 | 157 | Ref. |  |
| 11-15 | 1254 | 36 | 1419 | 5.4 | (2.5-11.4) | 11.4 | (3.8-34.2) | 36 | 1527 | 5.4 | (2.6-11.2) | 9.7 | (3.5-26.7) | 7 | 268 | Ref. |  |
| **Psychiatric medication** | |  |  |  |  |  |  |  |  |  |  |  |  |  |  |  | |
| All | 2556 | 239 | 5564 | 4.6 | (3.6-5.9) | 3.7 | (2.7-5.0) | 208 | 4995 | 4.0 | (3.1-5.1) | 3.2 | (2.4-4.4) | 66 | 1333 | Ref. |  |
| 6-10 | 1302 | 96 | 4313 | 4.9 | (3.3-7.3) | 3.6 | (2.3-5.9) | 80 | 3728 | 4.1 | (2.7-6.2) | 3.5 | (2.1-5.7) | 25 | 1021 | Ref. |  |
| 11-15 | 1254 | 143 | 6909 | 4.5 | (3.3-6.2) | 4.0 | (2.5-6.2) | 128 | 6342 | 3.9 | (2.9-5.4) | 3.4 | (2.2-5.1) | 41 | 1638 | Ref. |  |
| **Suicide attempt** | |  |  |  |  |  |  |  |  |  |  |  |  |  |  |  | |
| All | 2556 | 21 | 407 | 3.0 | (1.4-6.3) | 2.9 | (0.8-10.6) | 14 | 287 | 2.2 | (1.0-5.0) | 0.9 | (0.3-2.8) | 7 | 136 | Ref. |  |
| 6-10 | 1302 | 12 | 475 | 4.0 | (1.3-12.1) | N/A |  | 5 | 208 | 1.9 | (0.5-7.1) | N/A |  | 3 | 118 | Ref. |  |
| 11-15 | 1254 | 9 | 343 | 2.3 | (0.8-6.1) | N/A |  | 9 | 364 | 2.4 | (0.9-6.5) | N/A |  | 4 | 154 | Ref. |  |
| **Sick leave with psychiatric diagnosis**^2^ | |  |  |  |  |  |  |  |  |  |  |  |  |  |  |  | |
| 11-15 | 611 | 19 | 2721 | 5.0 | (1.9-12.8) | N/A |  | 8 | 1179 | 2.3 | (0.9-6.2) | N/A |  | 4 | 552 | Ref. |  |
| **Disability pension for any reason**^2^ | |  |  |  |  |  |  |  |  |  |  |  |  |  |  |  | |
| 11-15 | 611 | 20 | 2952 | 3.3 | (1.5-7.5) | N/A |  | 15 | 2341 | 2.6 | (1.1-6.3) | N/A |  | 6 | 849 | Ref. |  |
| **Age at start of follow-up** | **total N** | **N** | **IR** | **OR** | **95%CI** | **aOR^2^** | **95%CI** | **N** | **IR** | **OR** | **95%CI** | **aOR^2^** | **95%CI** | **N** | **IR** | **OR** |  |
| **Not completed secondary school in year when aged 19**^3^ | |  |  |  |  |  |  |  |  |  |  |  |  |  |  |  | |
| 11-15 | 611 | 73 |  | 2.2 | (1.4-3.4) | 1.7 | (0.9-3.2) | 67 |  | 2.4 | (1.5-4.0) | 1.9 | (0.9-3.8) | 42 |  | Ref. |  |

^1^Adjusted for parental history of substance use disorders, depression, other psychiatric disorders, self-harm/suicide attempt, non-psychiatric health care utilization, disability pension, sickness absence, family situation, number of children and country of birth, as well as offspring history of self-harm/suicide attempt, psychiatric diagnoses and non-psychiatric health care utilization. ^2^Only offspring for whom data is available from July 1 in the calendar year in which they reach 19 years of age. ^3^ Only offspring for whom data is available for the full calendar year in which they reach 19 years of age.
